# 1-[2-(3-Meth­oxy­phen­yl)eth­yl]pyrroli­dine-2,5-dione

**DOI:** 10.1107/S1600536813023751

**Published:** 2013-08-31

**Authors:** Zeenat Fatima, Jayaraman Selvakumar, Thothadri Srinivasan, Devadasan Velmurugan

**Affiliations:** a Centre of Advanced Study in Crystallography and Biophysics, University of Madras, Guindy Campus, Chennai 600 025, India; b Department of Chemistry, Pondicherry University, Puducherry 605 014, India

## Abstract

In the title compound, C_13_H_15_NO_3_, the pyrrolidine ring makes a dihedral angle of 4.69 (9)° with the 3-meth­oxy-phenyl ring. In the crystal, hydrogen-bonded chains running along [101] are generated by connecting neighbouring mol­ecules *via* C—H⋯O hydrogen bonds. Parallel chains are linked by further C—H⋯O hydrogen bonds, forming a three-dimensional structure.

## Related literature

For the bioactivity of pyrrolidine-2,5-dione derivatives, see: Obniska *et al.* (2012 ▶); Ha *et al.* (2011 ▶); Kaminski *et al.* (2011 ▶). For related structures, see: Khorasani & Fernandes (2012 ▶); Mayes *et al.* (2008 ▶).
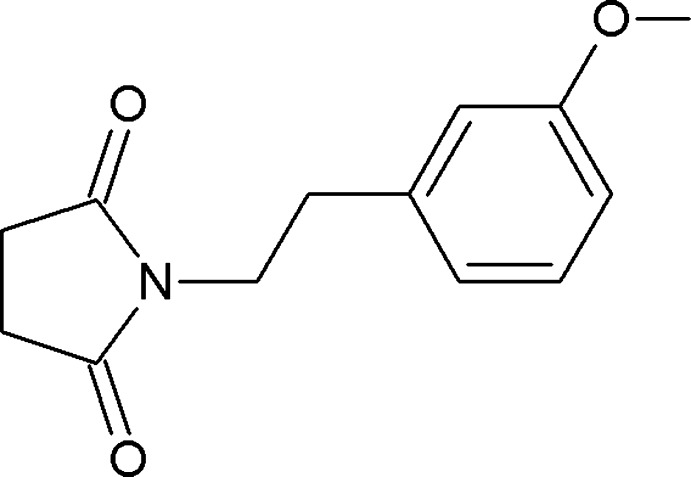


## Experimental

### 

#### Crystal data

C_13_H_15_NO_3_
*M* *_r_* = 233.26
Monoclinic, 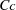

*a* = 12.8719 (9) Å
*b* = 12.5878 (8) Å
*c* = 7.4523 (5) Å
β = 90.831 (3)°
*V* = 1207.36 (14) Å^3^
*Z* = 4
Mo *K*α radiation
μ = 0.09 mm^−1^
*T* = 293 K
0.40 × 0.35 × 0.20 mm

#### Data collection

Bruker SMART APEXII area-detector diffractometer
Absorption correction: multi-scan (*SADABS*; Bruker, 2008 ▶) *T*_min_ = 0.964, *T*_max_ = 0.982
5692 measured reflections
2615 independent reflections
2328 reflections with *I* > 2σ(*I*)
*R*_int_ = 0.024

#### Refinement

*R*[*F* ^2^ > 2σ(*F* ^2^)] = 0.035
*wR*(*F* ^2^) = 0.097
*S* = 1.02
2615 reflections
156 parameters
2 restraints
H-atom parameters constrained
Δρ_max_ = 0.12 e Å^−3^
Δρ_min_ = −0.16 e Å^−3^

### 

Data collection: *APEX2* (Bruker, 2008 ▶); cell refinement: *SAINT* (Bruker, 2008 ▶); data reduction: *SAINT*; program(s) used to solve structure: *SHELXS97* (Sheldrick, 2008 ▶); program(s) used to refine structure: *SHELXL97* (Sheldrick, 2008 ▶); molecular graphics: *ORTEP-3 for Windows* (Farrugia, 2012 ▶); software used to prepare material for publication: *SHELXL97* and *PLATON* (Spek, 2009 ▶).

## Supplementary Material

Crystal structure: contains datablock(s) global, I. DOI: 10.1107/S1600536813023751/su2640sup1.cif

Structure factors: contains datablock(s) I. DOI: 10.1107/S1600536813023751/su2640Isup2.hkl

Supplementary material file. DOI: 10.1107/S1600536813023751/su2640Isup3.cml

Additional supplementary materials:  crystallographic information; 3D view; checkCIF report

## Figures and Tables

**Table 1:** Hydrogen-bond geometry (Å, °)

*D*—H⋯*A*	*D*—H	H⋯*A*	*D*⋯*A*	*D*—H⋯*A*
C1—H1*B*⋯O3^i^	0.96	2.54	3.418 (3)	151
C8—H8*B*⋯O1^ii^	0.97	2.54	3.469 (2)	161
C12—H12*A*⋯O2^iii^	0.97	2.57	3.456 (3)	152

Symmetry codes: (i) 
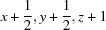
; (ii) 
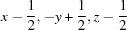
; (iii) 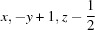.

**Fig. 1.**
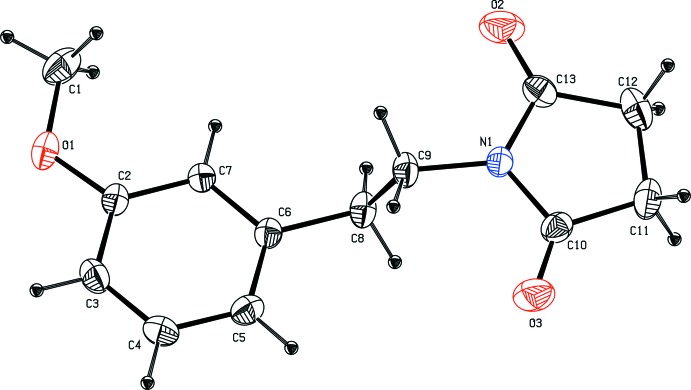
The molecular structure of the title molecule, with atom labelling. Displacement ellipsoids are drawn at the 30% probability level.

**Fig. 2.**
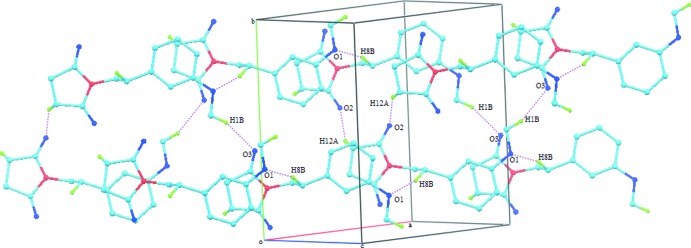
The crystal packing of the title compound viewed along the c axis. Hydrogen bonds are shown as dashed lines (see Table 1 for details; H atoms not involved in hydrogen bonding have been omitted for clarity).

## supplementary crystallographic information

### 1. Comment

Pyrrolidine-2,5-dione derivates are an important class of heterocylic compounds with essential applications in medicinal chemistry and organic synthesis. They exhibit numerous bioactivities, for example anticonvulsant (Obniska *et al.*, 2012; Kaminski *et al.*, 2011) and tyrosinase inhibitory activity (Ha *et al.*, 2011). In the field of organic chemistry derivates, like 1-bromopyrrolidine-2,5-dione (NBS), are the most commonly used halogenation reagents. In view of the different applications of this class of compounds, we have synthesized the title derivative and report herein on its crystal structure.

In the title compound, Fig. 1, the pyrrolidine ring (N1/C10—C13) makes a dihedral angle of 4.69 (9)° with the benzene ring (C2—C7).

In the crystal, hydrogen-bonded chains running along [101] are generated by connecting neighbouring molecules *via* C—H···O hydrogen bonds (Table 1 and Fig. 2). Parallel chains are linked by further C—H···O hydrogen bonds forming a three-dimensional structure (Table 1 and Fig. 2).

### 2. Experimental

3-methoxy phenethylamine (1.51 g, 10 mmol) and succinic anhydride (1.2 g, 12 mmol) were stirred at room temperature in dry ethyl acetate for 30 min. Ethyl acetate was removed under reduced pressure, and the resulting residue was dissolved in toluene. Acetyl chloride (5 equiv) was then added and the mixture refluxed for 1 h. The reaction mixture was washed with aqueous Na_2_CO_3_ and dried over anhydrous Na_2_SO_4_, filtered and concentrated under reduced pressure followed by silica gel column purification using hexane ethyl acetate (30:70) as eluent to afford the title compound as a colourless solid. Single crystals suitable for X-ray diffraction analysis were obtained by slow evaporation of a solution of the title compound in ethanol at room temperature.

### 3. Refinement

The H atoms were placed in calculated positions and treated as riding atoms: C—H = 0.93 Å to 0.97 Å, with *U*_iso_(H) = 1.5Ueq(C-methyl) and = 1.2Ueq(C) for other H atoms.

### Crystal data

**Table:**

C_13_H_15_NO_3_	*F*(000) = 496
*M**_r_* = 233.26	*D*_x_ = 1.283 Mg m^−^^3^
Monoclinic, *C**c*	Mo *K*α radiation, λ = 0.71073 Å
Hall symbol: C -2yc	Cell parameters from 2615 reflections
*a* = 12.8719 (9) Å	θ = 2.3–28.4°
*b* = 12.5878 (8) Å	µ = 0.09 mm^−^^1^
*c* = 7.4523 (5) Å	*T* = 293 K
β = 90.831 (3)°	Block, colourless
*V* = 1207.36 (14) Å^3^	0.40 × 0.35 × 0.20 mm
*Z* = 4	

### Data collection

**Table:**

Bruker SMART APEXII area-detector diffractometer	2615 independent reflections
Radiation source: fine-focus sealed tube	2328 reflections with *I* > 2σ(*I*)
Graphite monochromator	*R*_int_ = 0.024
ω and φ scans	θ_max_ = 28.4°, θ_min_ = 2.3°
Absorption correction: multi-scan (*SADABS*; Bruker, 2008)	*h* = −17→17
*T*_min_ = 0.964, *T*_max_ = 0.982	*k* = −15→16
5692 measured reflections	*l* = −9→9

### Refinement

**Table:**

Refinement on *F*^2^	Secondary atom site location: difference Fourier map
Least-squares matrix: full	Hydrogen site location: inferred from neighbouring sites
*R*[*F*^2^ > 2σ(*F*^2^)] = 0.035	H-atom parameters constrained
*wR*(*F*^2^) = 0.097	*w* = 1/[σ^2^(*F*_o_^2^) + (0.0544*P*)^2^ + 0.1677*P*] where *P* = (*F*_o_^2^ + 2*F*_c_^2^)/3
*S* = 1.02	(Δ/σ)_max_ < 0.001
2615 reflections	Δρ_max_ = 0.12 e Å^−^^3^
156 parameters	Δρ_min_ = −0.16 e Å^−^^3^
2 restraints	Extinction correction: *SHELXL*, Fc^*^=kFc[1+0.001xFc^2^λ^3^/sin(2θ)]^-1/4^
Primary atom site location: structure-invariant direct methods	Extinction coefficient: 0.0149 (15)

### Special details

**Table:**

Geometry. All e.s.d.'s (except the e.s.d. in the dihedral angle between two l.s. planes) are estimated using the full covariance matrix. The cell e.s.d.'s are taken into account individually in the estimation of e.s.d.'s in distances, angles and torsion angles; correlations between e.s.d.'s in cell parameters are only used when they are defined by crystal symmetry. An approximate (isotropic) treatment of cell e.s.d.'s is used for estimating e.s.d.'s involving l.s. planes.
Refinement. Refinement of *F*^2^ against ALL reflections. The weighted *R*-factor *wR* and goodness of fit *S* are based on *F*^2^, conventional *R*-factors *R* are based on *F*, with *F* set to zero for negative *F*^2^. The threshold expression of *F*^2^ > σ(*F*^2^) is used only for calculating *R*-factors(gt) *etc*. and is not relevant to the choice of reflections for refinement. *R*-factors based on *F*^2^ are statistically about twice as large as those based on *F*, and *R*- factors based on ALL data will be even larger.

### Fractional atomic coordinates and isotropic or equivalent isotropic displacement parameters (Å^2^)

**Table:**

	*x*	*y*	*z*	*U*_iso_*/*U*_eq_	
C1	0.95763 (19)	0.43725 (18)	1.0655 (3)	0.0832 (6)	
H1A	0.9806	0.4494	0.9452	0.125*	
H1B	1.0043	0.4716	1.1487	0.125*	
H1C	0.8890	0.4657	1.0789	0.125*	
C2	0.90109 (11)	0.26275 (13)	0.9852 (2)	0.0485 (3)	
C3	0.91325 (14)	0.15484 (15)	1.0131 (2)	0.0633 (4)	
H3	0.9582	0.1300	1.1025	0.076*	
C4	0.85800 (17)	0.08476 (14)	0.9071 (3)	0.0695 (5)	
H4	0.8652	0.0121	0.9261	0.083*	
C5	0.79213 (14)	0.12096 (13)	0.7730 (2)	0.0609 (4)	
H5	0.7552	0.0726	0.7026	0.073*	
C6	0.78069 (11)	0.22844 (13)	0.74273 (18)	0.0472 (3)	
C7	0.83541 (11)	0.29992 (12)	0.85029 (19)	0.0450 (3)	
H7	0.8280	0.3726	0.8317	0.054*	
C8	0.70939 (12)	0.26805 (15)	0.5942 (2)	0.0564 (4)	
H8A	0.6893	0.3408	0.6193	0.068*	
H8B	0.6468	0.2251	0.5907	0.068*	
C9	0.76102 (13)	0.26346 (15)	0.4132 (2)	0.0565 (4)	
H9A	0.8161	0.3159	0.4094	0.068*	
H9B	0.7920	0.1939	0.3972	0.068*	
C10	0.63151 (13)	0.20320 (14)	0.1850 (2)	0.0573 (4)	
C11	0.56115 (15)	0.25110 (19)	0.0462 (3)	0.0737 (6)	
H11A	0.4891	0.2352	0.0712	0.088*	
H11B	0.5774	0.2247	−0.0724	0.088*	
C12	0.58151 (16)	0.36961 (19)	0.0592 (3)	0.0777 (6)	
H12A	0.6066	0.3969	−0.0540	0.093*	
H12B	0.5185	0.4073	0.0896	0.093*	
C13	0.66250 (15)	0.38270 (13)	0.2046 (2)	0.0609 (4)	
N1	0.68700 (9)	0.28356 (10)	0.26754 (15)	0.0475 (3)	
O1	0.95629 (10)	0.32628 (11)	1.10049 (17)	0.0690 (3)	
O2	0.70187 (17)	0.46337 (11)	0.2584 (2)	0.0967 (5)	
O3	0.64082 (15)	0.11124 (11)	0.2245 (2)	0.0906 (5)	

### Atomic displacement parameters (Å^2^)

**Table:**

	*U*^11^	*U*^22^	*U*^33^	*U*^12^	*U*^13^	*U*^23^
C1	0.0866 (14)	0.0787 (13)	0.0843 (14)	−0.0200 (11)	−0.0031 (11)	−0.0264 (11)
C2	0.0412 (7)	0.0623 (8)	0.0421 (7)	0.0004 (7)	0.0000 (6)	−0.0038 (7)
C3	0.0629 (10)	0.0683 (10)	0.0585 (10)	0.0098 (8)	−0.0075 (8)	0.0138 (8)
C4	0.0825 (12)	0.0484 (9)	0.0773 (12)	0.0001 (8)	−0.0032 (10)	0.0119 (8)
C5	0.0636 (10)	0.0534 (9)	0.0658 (11)	−0.0099 (7)	−0.0003 (8)	−0.0074 (7)
C6	0.0405 (7)	0.0586 (8)	0.0425 (7)	−0.0008 (6)	0.0019 (6)	−0.0018 (6)
C7	0.0429 (7)	0.0486 (7)	0.0437 (7)	0.0016 (6)	0.0014 (6)	−0.0002 (6)
C8	0.0444 (7)	0.0769 (10)	0.0478 (8)	0.0049 (7)	−0.0064 (6)	−0.0044 (8)
C9	0.0434 (7)	0.0776 (11)	0.0483 (8)	0.0044 (7)	−0.0084 (6)	0.0034 (8)
C10	0.0570 (9)	0.0649 (11)	0.0502 (8)	−0.0133 (7)	0.0058 (7)	−0.0053 (7)
C11	0.0508 (9)	0.1229 (19)	0.0473 (8)	−0.0130 (10)	−0.0052 (7)	0.0007 (10)
C12	0.0687 (12)	0.1047 (16)	0.0596 (10)	0.0264 (11)	0.0000 (9)	0.0204 (10)
C13	0.0717 (11)	0.0584 (9)	0.0529 (10)	0.0049 (8)	0.0093 (8)	0.0062 (7)
N1	0.0448 (6)	0.0538 (7)	0.0439 (7)	−0.0009 (5)	−0.0041 (5)	−0.0004 (5)
O1	0.0654 (7)	0.0838 (9)	0.0573 (7)	−0.0055 (6)	−0.0185 (5)	−0.0104 (6)
O2	0.1454 (15)	0.0532 (7)	0.0917 (10)	−0.0170 (9)	0.0081 (10)	−0.0025 (7)
O3	0.1184 (13)	0.0569 (8)	0.0962 (12)	−0.0184 (7)	−0.0033 (10)	−0.0054 (7)

### Geometric parameters (Å, º)

**Table:**

C1—O1	1.421 (3)	C8—H8A	0.9700
C1—H1A	0.9600	C8—H8B	0.9700
C1—H1B	0.9600	C9—N1	1.4562 (19)
C1—H1C	0.9600	C9—H9A	0.9700
C2—O1	1.3654 (19)	C9—H9B	0.9700
C2—C3	1.383 (2)	C10—O3	1.200 (2)
C2—C7	1.386 (2)	C10—N1	1.378 (2)
C3—C4	1.376 (3)	C10—C11	1.492 (3)
C3—H3	0.9300	C11—C12	1.517 (3)
C4—C5	1.379 (3)	C11—H11A	0.9700
C4—H4	0.9300	C11—H11B	0.9700
C5—C6	1.379 (2)	C12—C13	1.502 (3)
C5—H5	0.9300	C12—H12A	0.9700
C6—C7	1.390 (2)	C12—H12B	0.9700
C6—C8	1.512 (2)	C13—O2	1.201 (2)
C7—H7	0.9300	C13—N1	1.368 (2)
C8—C9	1.513 (2)		
			
O1—C1—H1A	109.5	H8A—C8—H8B	107.9
O1—C1—H1B	109.5	N1—C9—C8	111.51 (13)
H1A—C1—H1B	109.5	N1—C9—H9A	109.3
O1—C1—H1C	109.5	C8—C9—H9A	109.3
H1A—C1—H1C	109.5	N1—C9—H9B	109.3
H1B—C1—H1C	109.5	C8—C9—H9B	109.3
O1—C2—C3	115.10 (14)	H9A—C9—H9B	108.0
O1—C2—C7	124.41 (14)	O3—C10—N1	123.31 (18)
C3—C2—C7	120.48 (14)	O3—C10—C11	128.13 (18)
C4—C3—C2	119.16 (15)	N1—C10—C11	108.56 (15)
C4—C3—H3	120.4	C10—C11—C12	104.51 (15)
C2—C3—H3	120.4	C10—C11—H11A	110.8
C3—C4—C5	120.78 (16)	C12—C11—H11A	110.9
C3—C4—H4	119.6	C10—C11—H11B	110.8
C5—C4—H4	119.6	C12—C11—H11B	110.9
C4—C5—C6	120.39 (16)	H11A—C11—H11B	108.9
C4—C5—H5	119.8	C13—C12—C11	105.73 (15)
C6—C5—H5	119.8	C13—C12—H12A	110.6
C5—C6—C7	119.25 (14)	C11—C12—H12A	110.6
C5—C6—C8	120.36 (14)	C13—C12—H12B	110.6
C7—C6—C8	120.39 (15)	C11—C12—H12B	110.6
C2—C7—C6	119.91 (14)	H12A—C12—H12B	108.7
C2—C7—H7	120.0	O2—C13—N1	124.25 (18)
C6—C7—H7	120.0	O2—C13—C12	128.20 (19)
C6—C8—C9	111.72 (12)	N1—C13—C12	107.54 (16)
C6—C8—H8A	109.3	C13—N1—C10	113.65 (14)
C9—C8—H8A	109.3	C13—N1—C9	123.99 (15)
C6—C8—H8B	109.3	C10—N1—C9	122.31 (15)
C9—C8—H8B	109.3	C2—O1—C1	117.90 (14)
			
O1—C2—C3—C4	177.92 (17)	C10—C11—C12—C13	0.1 (2)
C7—C2—C3—C4	−1.1 (3)	C11—C12—C13—O2	179.5 (2)
C2—C3—C4—C5	0.8 (3)	C11—C12—C13—N1	0.5 (2)
C3—C4—C5—C6	0.1 (3)	O2—C13—N1—C10	−179.95 (18)
C4—C5—C6—C7	−0.7 (3)	C12—C13—N1—C10	−0.94 (19)
C4—C5—C6—C8	179.27 (16)	O2—C13—N1—C9	2.7 (3)
O1—C2—C7—C6	−178.43 (15)	C12—C13—N1—C9	−178.33 (15)
C3—C2—C7—C6	0.4 (2)	O3—C10—N1—C13	−178.52 (19)
C5—C6—C7—C2	0.5 (2)	C11—C10—N1—C13	1.00 (19)
C8—C6—C7—C2	−179.55 (14)	O3—C10—N1—C9	−1.1 (2)
C5—C6—C8—C9	−80.96 (19)	C11—C10—N1—C9	178.43 (16)
C7—C6—C8—C9	99.05 (18)	C8—C9—N1—C13	87.3 (2)
C6—C8—C9—N1	169.92 (15)	C8—C9—N1—C10	−89.88 (19)
O3—C10—C11—C12	178.89 (19)	C3—C2—O1—C1	172.27 (18)
N1—C10—C11—C12	−0.6 (2)	C7—C2—O1—C1	−8.8 (2)

### Hydrogen-bond geometry (Å, º)

**Table:**

*D*—H···*A*	*D*—H	H···*A*	*D*···*A*	*D*—H···*A*
C1—H1*B*···O3^i^	0.96	2.54	3.418 (3)	151
C8—H8*B*···O1^ii^	0.97	2.54	3.469 (2)	161
C12—H12*A*···O2^iii^	0.97	2.57	3.456 (3)	152

Symmetry codes: (i) *x*+1/2, *y*+1/2, *z*+1; (ii) *x*−1/2, −*y*+1/2, *z*−1/2; (iii) *x*, −*y*+1, *z*−1/2.
